# Impact of cyclosporine A on the progression of ocular surface diseases: treatment efficacy and potential complications

**DOI:** 10.1099/jmm.0.001978

**Published:** 2025-03-12

**Authors:** Gulnara Begimbayeva, Tursunkul Botabekova, Assel Yelikbayeva, Ekaterina Voronkova, Kamilla Kenzhebayeva

**Affiliations:** 1 Department of Ophthalmology, Kazakh-Russian Medical University, 71 Torekulov Str., 050000, Almaty, Republic of Kazakhstan; 2 Department of Ophthalmology, Ophthalmological Centre "FOCUS", 450 Seifullin Ave., 050000, Almaty, Republic of Kazakhstan

**Keywords:** conjunctiva, dysfunctional tear syndrome, inflammation, Meibomian gland, Schirmer test

## Abstract

**Introduction.** Eye diseases are widespread all over the world and, if left untreated, can lead to blindness.

**Hypothesis.** The use of 0.05% cyclosporine A (CsA) solution for the treatment of dry eye causes a decrease in discomfort and pain and improves objective measures such as tear film breakdown time, Schirmer test results and Oxford scale scores due to its anti-inflammatory and immunomodulatory properties that contribute to improved tear film stability and tear production.

**Aim.** This study aimed to investigate the impact of CsA on the progression of ocular surface diseases.

**Methodology.** An experiment was conducted on the basis of the FOCUS ophthalmology centre, Kazakhstan. A group of 100 individuals diagnosed with mild to severe dry eye illness were administered 0.05% CsA eye drops as part of the treatment protocol.

**Results.** The positive effect is explained by the anti-inflammatory and immunomodulatory effects of CsA. The study showed that the use of 0.05% CsA solution for the treatment of ocular surface disease led to a decrease in discomfort and pain, as well as an improvement in objective measures, including tear film breakdown time, Schirmer test results and Oxford scale scores. The visual analogue scale showed a significant reduction in symptoms, from 6.8 points at baseline to 3.7 points at day 60. In total, 20% of patients reported moderate side effects, such as pain during instillation and redness of the eye surface.

**Conclusion.** It is concluded that the use of these drops reduced pain, improved patient condition and enhanced indicators such as the visual analogue scale, tear film breakup time, Schirmer test and Oxford scale of corneal and conjunctival staining. This study differs from the previous ones in that it focuses on the efficacy of 0.05% CsA solution in the treatment of dry eye and also examines the spectrum and frequency of side effects, which is not sufficiently covered in previous studies.

## Introduction

Around 253 million individuals suffer from visual impairments, with over 36 million of them having full blindness [1]. The rise in life expectancy and subsequent population ageing have led to a rise in the prevalence of cataracts and glaucoma. Additionally, the continuous and prolonged use of tablets and computers has contributed to an increase in the occurrence of myopia. Furthermore, the frequent use of digital devices and prolonged exposure to dry air has increased the number of cases of dry eye syndrome. Notably, eye diseases negatively affect the daily activities of patients, the degree of productivity, daily habits and physical and social activity.

Dry eye disease or dry keratoconjunctivitis is estimated to be between 5 and 50%, with more frequent female lesions [1]. Most often, 0.05% cyclosporine A (CsA) eye drops are used for treatment, which causes inhibition of the calcineurin-phosphatase pathway due to the formation of an intracellular complex with cyclophilin. Clinically, this is manifested by the activation of natural lacrimation and an increase in the density of goblet cells. However, today, there are many pharmacological forms of CsA preparations on the market with varying degrees of digestibility, benefits and frequency of side effects; there is also a debate about the effectiveness of using only CsA eye drops in dry eye disease. Many Kazakh and foreign researchers have already considered the effectiveness of using CsA eye drops in combination with other medications for eye diseases and estimated the prevalence of many eye diseases among residents of Kazakhstan.

For example, researchers S.U. Kamenova *et al*. [2] evaluated the risks and incidence of non-motor vision disorders among elderly patients with Parkinson’s disease in Almaty. The prevalence of refractive errors and risk factors for myopia among schoolchildren in Almaty, Kazakhstan, was established by A. Mukazhanova *et al*. [3]. Researchers A. Kabylbekova *et al*. [4] investigated the characteristics of congenital cataracts and cataracts that develop in children under 10 years of age. The visual system of students residing in the southern region of Kazakhstan was observed by Karabalayeva *et al*. [5]. The impact of CsA on atypical sensory nerve activity, which is a defining feature of dry eye illness, was assessed by Gyenes *et al*. [6]. The article by D.D. Nguyen and J.-Y. Lai [7] explores the development of polymeric drug delivery systems that respond to stimuli to improve the treatment of eye diseases. The authors analyse the latest approaches to creating such systems that can change their properties in response to specific biological or physical stimuli, which allows for more accurate and efficient drug dosage. The study demonstrated that the use of polymeric systems sensitive to external or internal stimuli can significantly improve localized drug delivery to eye tissues, reducing side effects and improving therapeutic outcomes. Researchers D. Gao *et al*. [8] compared the efficacy, bioavailability and side effects of available registered CsA pharmacological agents for treating dry eye disease. Despite the extensive study of eye diseases, the works of the presented researchers did not study the effectiveness of topical application of only 0.05% CsA drops in dry eye disease. In addition, the literature describes cases of certain side effects due to the use of this drug.

Therefore, the study aimed to investigate the impact of CsA on the progression of ocular surface diseases. The objectives of the presented study were to examine the available literature on eye surface diseases and their treatment and analyse the spectrum and frequency of possible complications from the use of CsA.

## Methods

This is an open prospective study. Patient examinations and clinical trials were conducted at the FOCUS ophthalmology centre. The centre has modern equipment that allows performing modern diagnostic and therapeutic measures (computer vision diagnostics, optical biometrics, laser vision correction, keratotopography, optical coherent 3D tomography and surgical treatment of common eye pathologies). The centre employs highly qualified specialists with many years of experience.

The study had a sample size of 100 patients. The study participants were of legal age and capable; they signed a written informed consent to the processing of medical data, conducting diagnostic and therapeutic measures and participating in the presented study. The study encompassed a group of individuals ranging in age from 18 to 70 years, exhibiting varying degrees of dry eye disease severity. The exclusion criteria were as follows: severe degree of dry eye disease; surgical interventions on the eye during the last 10 months; surgical treatment of dry eye disease in the anamnesis; the presence of cataracts and glaucoma; the inability to refuse to wear contact lenses for the duration of the study; the presence of scars or the consequences of a chemical burn on the conjunctiva; taking replacement hormonal agents that cannot be abandoned for the duration of the study; severe uncompensated cardiovascular and mental disorders; individual intolerance to CsA or pharmacological agents used to diagnose dry eye disease.

Before starting the experiment, the selected participants underwent diagnostic tests. The Schirmer rupture test was performed without anaesthesia [9]. The test involved the use of paper strips positioned inside the region encompassing the central and lateral aspects of the lower eyelid while the eyes were in a closed position. The test results indicated dry eye syndrome if, after 5 min, the paper strip was moistened by less than 10 mm. The time of destruction of the tear film was determined (after the instillation of 0.2% fluorescein solution into the patient’s eye, the time of destruction of the tear film was determined biomicroscopically; it normally ranges from 10 s) [10]. The degree of pain and discomfort was assessed using the visual analogue scale, with 0 indicating the absence of symptoms and 10 indicating the most severe presentation of the disease [11]. Corneal and conjunctival colouring was examined on the Oxford scale after lissamine green staining [12]. The scale is represented by rows of panels, which are indicated by the letters from A to E, in ascending order of severity of the disease. The Oxford scale is used to classify damage to the surface cells of the eye by comparing the colour of the eye with reference panels labelled A to E, in order of increasing severity. The score starts with A, which indicates mild redness or minor damage to the surface of the eye without significant pathology. This is followed by B, which indicates mild damage with slight discolouration or minor irritation. C on this scale indicates moderate damage, with severe redness and small defects on the surface of the eye. D indicates severe damage, with large defects or erosions on the surface of the eye. Finally, E indicates the most severe damage, which may include large erosions or ulcers, significant redness and impaired eye function. The study recorded an improvement in this scale, indicating a reduction in ocular surface damage in patients treated with CsA.

The results of diagnostic tests were analysed and recorded. These tests were repeated on days 21 and 60 of the study. The participants also completed a questionnaire about how many hours a day they spend using digital equipment (phone, tablet or computer). Prior to the experiment, participants were offered training on the specifics of using CsA. Also, all patients were informed about the need to keep a diary and note their general state of health in it. If any atypical symptoms or adverse reactions occur, participants should immediately inform their doctor and consult over the phone to resolve the situation further. All participants were prescribed 0.05% CsA eye drops (one drop two times a day in the morning and evening) during the study period. All interventions were performed on a commercial basis and funded by patients or their insurance institutions according to the price list of the FOCUS ophthalmology centre, Kazakhstan.

In order to validate the results, statistical tools such as the Schirmer test, Oxford scale and visual analogue scale were utilized to assess the efficacy of 0.05% CsA drops in treating dry eye disease. These clinical tools provided significant evidence of the drug’s impact on tear production, ocular surface health and symptom reduction. In the graphs, the *x*-axis represents the time points of the study (i.e. Day 0, Day 21 and Day 60), while the *y*-axis indicates the respective measurements for each test, such as tear production (mm), Oxford scale indicators, visual analogue scale points and tear film breakdown time (s). The data demonstrated clear improvements across all measures, supporting the positive effects of CsA. Additionally, comparisons with other cyclosporine formulations, such as Restasis, Zirun and Ikervis, further reinforced the drug’s effectiveness and safety while highlighting its superior performance in specific parameters like the Schirmer test.

The available literature in the PubMed, EMBASE and Google Scholar databases published in 2018–2023 were analysed to better examine the features of CsA. A large-scale statistical analysis of the conducted studies was performed. The obtained data were analysed using the Matlab R2023a numerical software and its statistical tools.

Patients with severe dry eye were excluded from the study because this degree of disease requires a different approach to treatment and may have significant complications that affect the effectiveness of the use of 0.05% CsA solution. Excluding such patients avoids potentially distorted study results and provides a more accurate assessment of treatment efficacy among patients with mild to moderate dry eye, where the effect of the drug may be most pronounced. This also allows us to limit the application of the study results to these categories of patients only, where the therapeutic effect may be most obvious and measurable.

## Results

### The investigation results about the impact of 0.05% CsA drops on the progression of dry eye disease

**Fig. 1.**
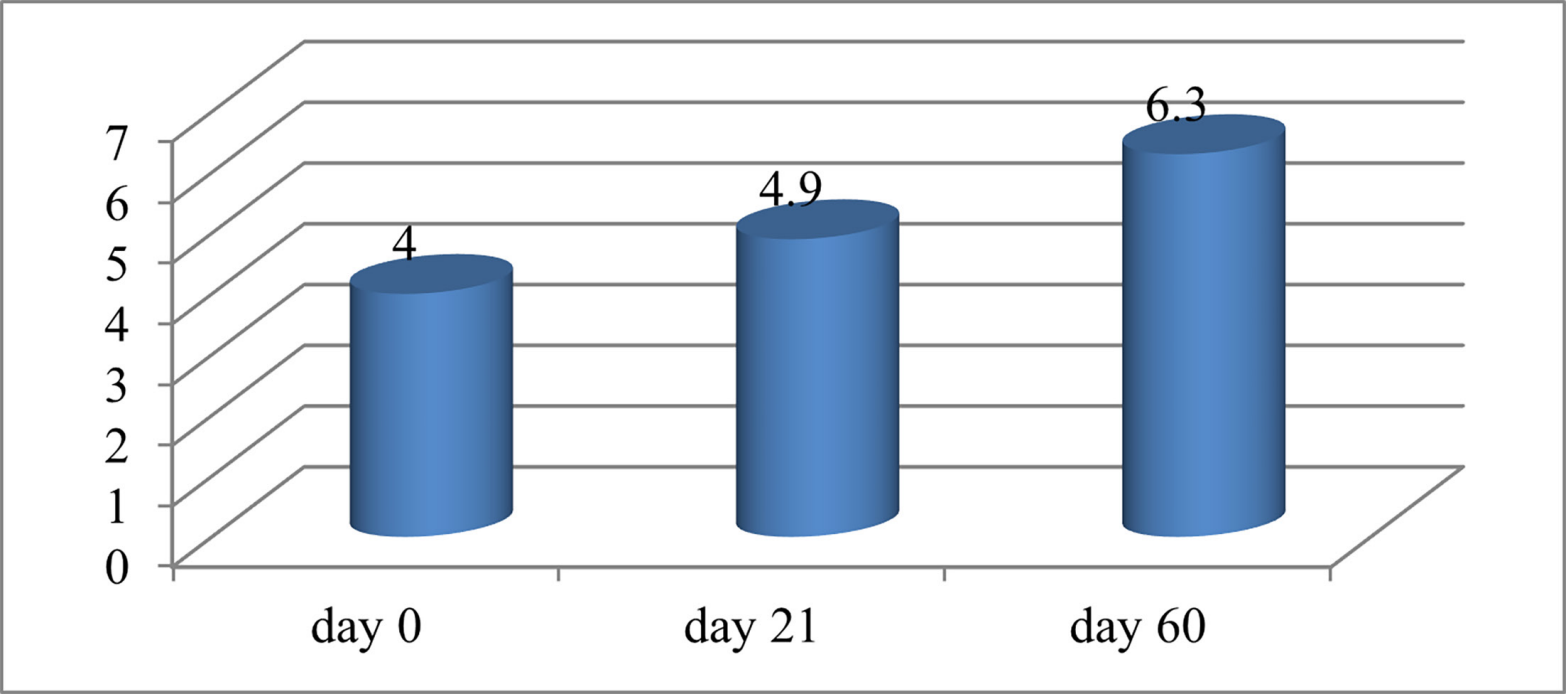
Average value of Schirmer test scores, millimetres.

The research encompassed a sample size of 100 individuals. The research participants had an average age of 51.6 years. The proportion of women was 52% (52 patients), while males constituted 48% (48 patients). At the beginning of the study, 60% (60 patients) of participants were diagnosed with mild dry eye disease and 40% (40 patients) with moderate severity. The values presented in Fig. 1 represent the average Schirmer test scores, which measure the amount of tear production in millimetres. The significant impact of CsA on tear production can be attributed to the Schirmer test, a technique used to assess the production of the water component of the tear film.

**Fig. 2.**
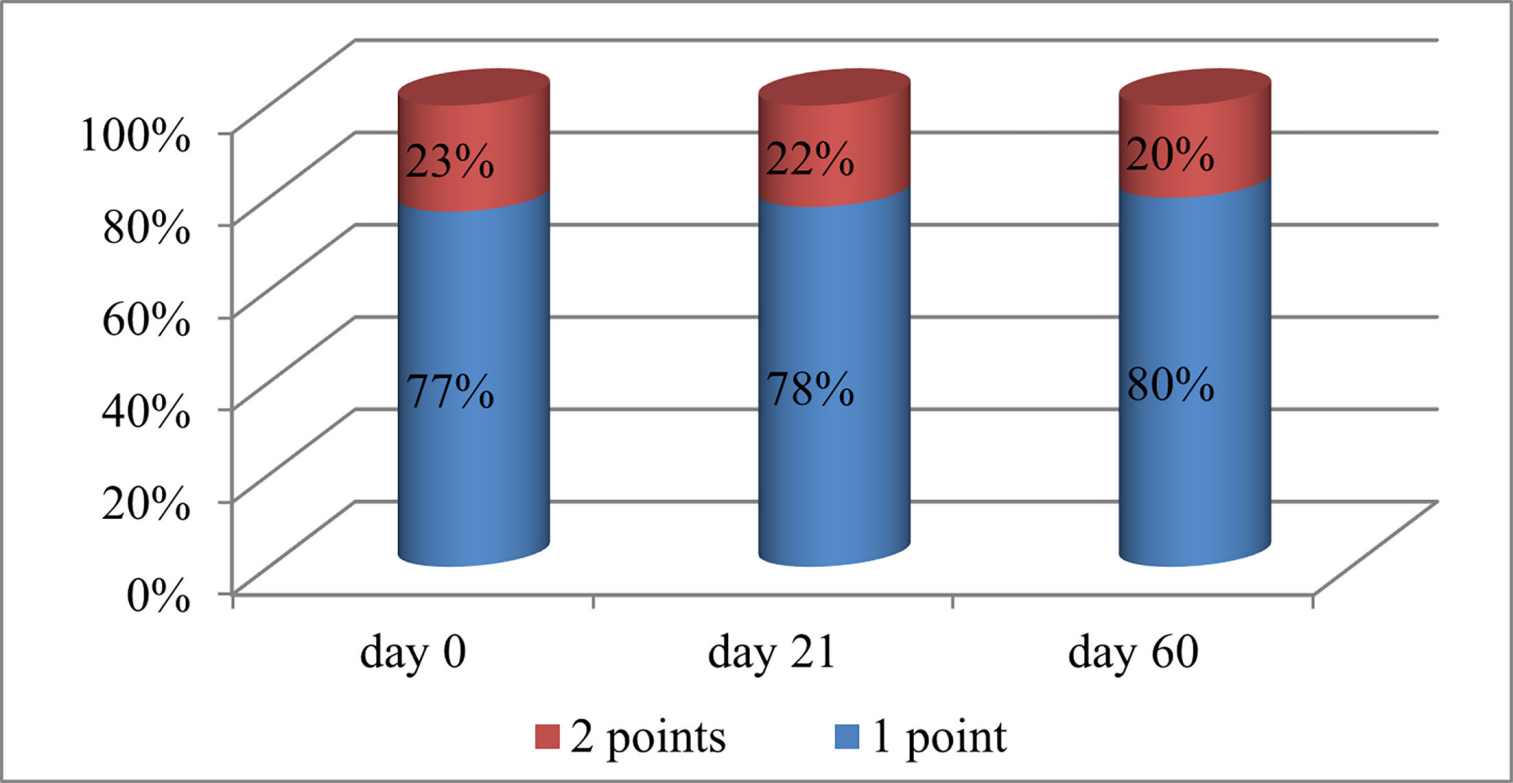
Oxford scale indicators. *Note:* ‘1 point’ (blue) refers to a lower severity of eye surface damage; ‘2 points’ (red) indicates a greater severity of the disease.

The Oxford scale is used to classify damage to the surface cells of the eye by comparing the colour in the eyes and standard panels marked from A to E, in ascending order of severity of the disease; as a result of the study, an improvement in indicators on this scale was obtained (Fig. 2).

**Fig. 3.**
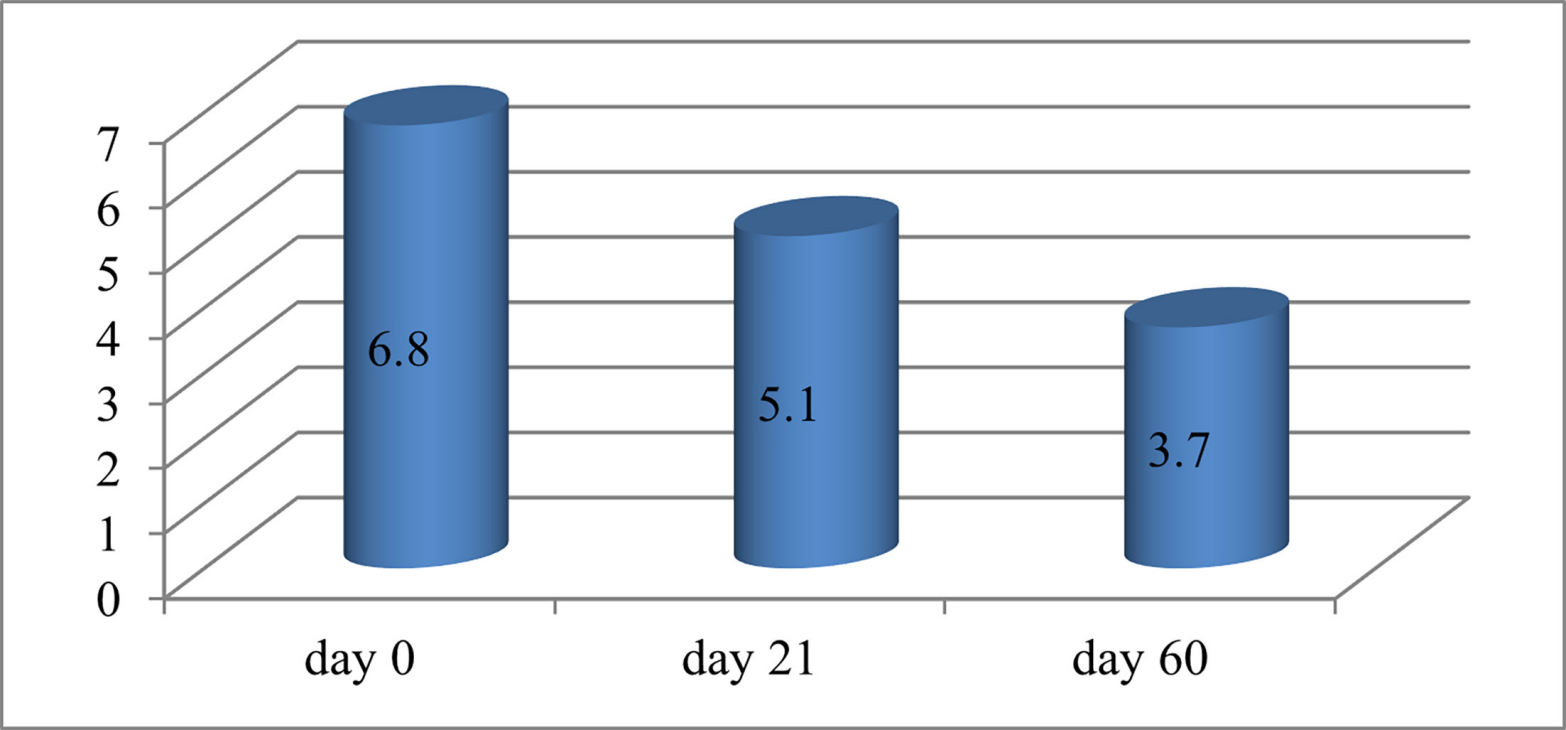
Results on a visually analogue scale, points.

When evaluated on a visually analogue scale, patients at the beginning of the study complained of a burning sensation, itching, severe discomfort, photophobia and pain. As a result of the study, there was an improvement in the general condition of patients on days 21 and 60 (Fig. 3). This is explained by the anti-inflammatory effect of CsA drops.

**Fig. 4.**
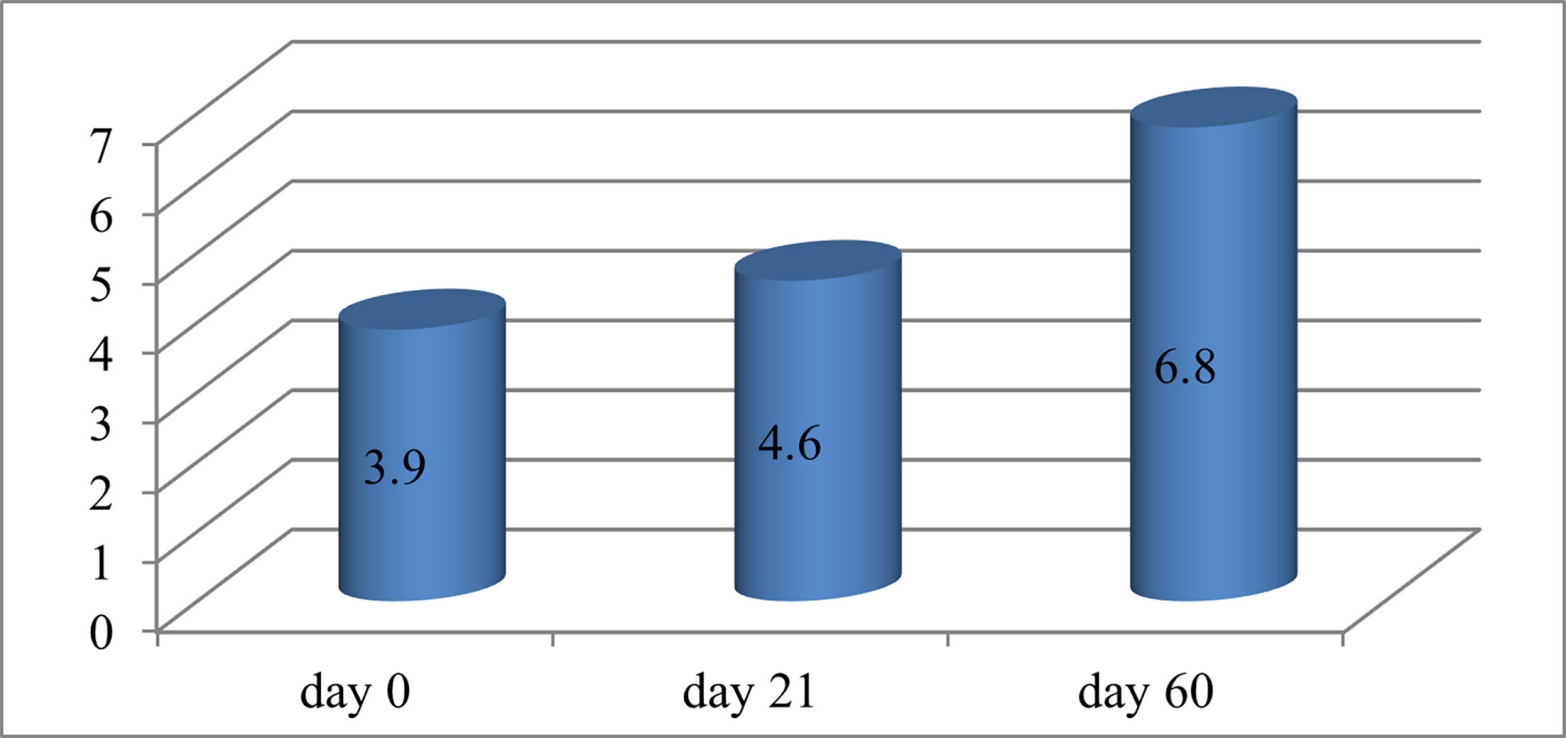
Tear film breakdown time, seconds.

Fig. 4 illustrates the tear film’s destruction time measured in seconds across different time points. The values (3.9, 4.6 and 6.8) indicate the measured tear film breakdown time at each respective time point, with an improvement observed over time. This is due to the ability of CsA to improve the density of goblet cells on the conjunctival surface.

**Fig. 5.**
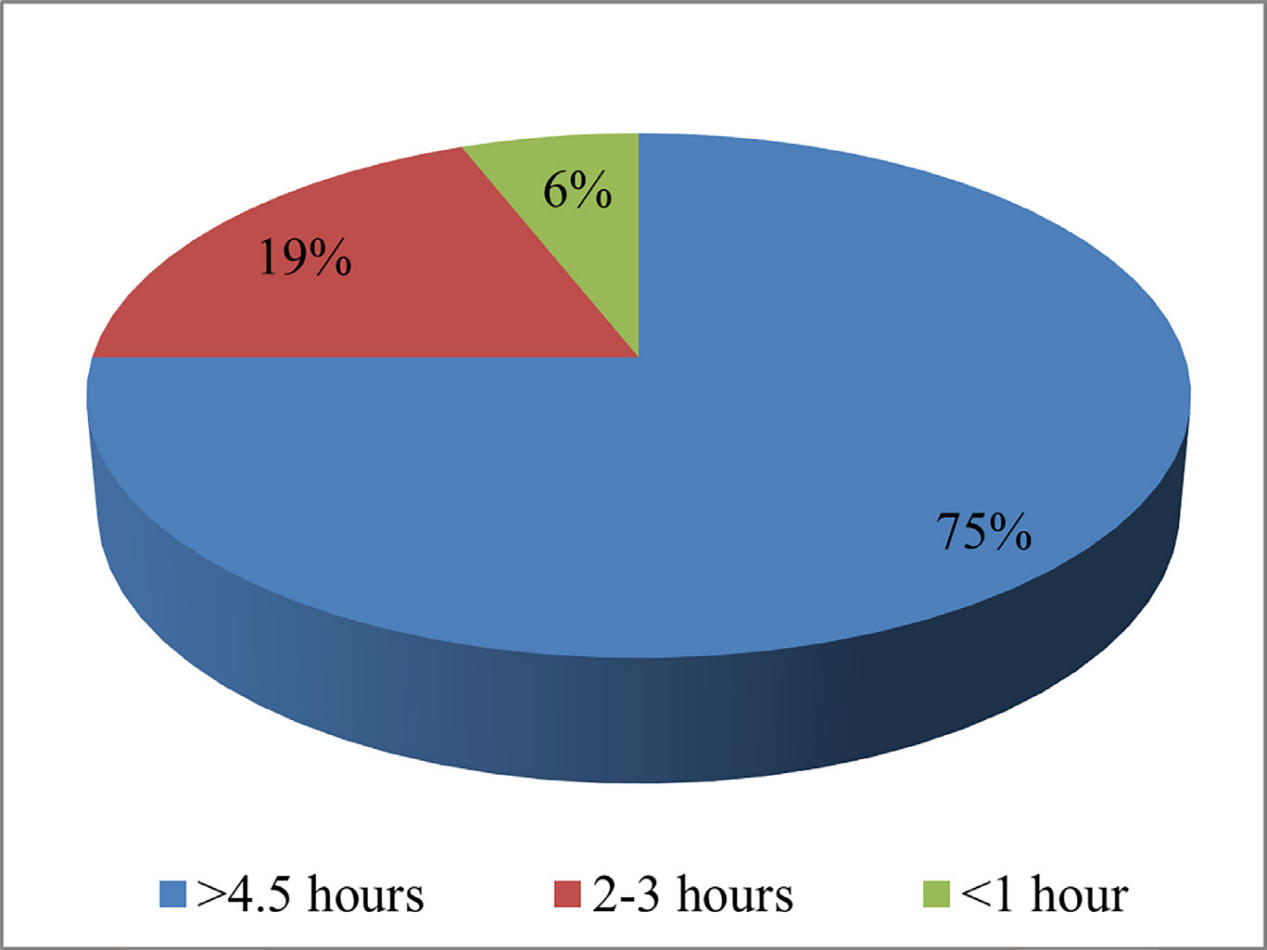
Distribution of survey responses regarding the daily usage of digital equipment.

The overwhelming majority of patients in the questionnaire completed before the study noted that they use digital equipment for more than 4.5 h a day (Fig. 5). The aforementioned element poses a risk for the development of dry eye illness.

In 20% of patients (15 women and 5 men) during the entire study period, complaints of moderate side effects were recorded: eye pain and burning sensation, tingling, pain during instillation of the eyes and periodic blurred vision. None of the patients had an allergic reaction or anaphylactic shock. All patients used prescribed eye drops during the entire study period.

### Clinical effects of CsA in dry eye disease

The human visual analyser is represented by the eyeball, the adnexal apparatus of the eye, the visual pathway, the central neuron and the visual centre. The eyeball itself consists of three membranes: fibrous, vascular and retinal. The surface structures of the eye include the cornea (this is the anterior part of the fibrous membrane, transparent and has a spherical shape); the conjunctiva (the ocular mucous membrane covers the back of the eyelids and the front of the sclera; at the point of transition of the conjunctiva from the eyelids to the eyeball, an arch is formed). The adnexal apparatus of the eye performs a protective function. It is represented by the upper and lower eyelids and lacrimal glands (located in the upper-outer part of the orbit, responsible for constant moistening of the cornea and conjunctiva).

Pathogenesis is based on a violation of the structure of the tear film and the ingress of pathogens. Normally, the tear consists of three layers. The inner (mucin) layer covers the cornea and protects it from excessive friction. The middle (water) layer consists of 98% water and 2% protein, cleanses the surface of the eye from harmful substances and provides nutrients and oxygen. The outer (lipid) layer is a fat film that prevents excessive evaporation of water [12,13]. With insufficient production of one of the components, the tear quickly evaporates, insufficiently moisturizes and protects the eye, which causes favourable conditions for the penetration and development of pathogenic factors. Due to the action of damaging exogenous or endogenous factors, the immune system of the eye surface is activated. The immune response develops on the surface of the cornea and is regulated by the corneal epithelium. Chemokines, T-killers, macrophages, cytokines and chemokines (TNF-a, IL-1 and IL-6) that stimulate antigen-presenting cell maturation are activated. Primary immune cells, known as mature antigen-presenting cells, are responsible for integrating both innate and acquired immune responses. These structures promote the differentiation of T-cells and their migration to the conjunctiva and eye surface.

The clinical manifestations of the disease depend on the severity of the disease. With a mild degree, excessive tear fluid is produced, and the tear film thickens along the posterior edge of the lower eyelid; patients complain of lacrimation, eye redness and blurred vision. The average degree is characterized by a reduced production of tear fluid; there is a thinning of the tear film along the posterior edge of the lower eyelid; there is a feeling of sand in the eyes, burning and pain. In severe cases, filamentous epithelial growths and erosions are observed on the cornea, and sharp pain and blurred vision occur. In the case of severely neglected processes, a rupture of the cornea is possible. It was examined that after cataract surgery, dry eye symptoms develop in 40, 15 and 10% of patients after 7, 30 and 90 days, respectively [14,15]. It was examined that CsA is an effective anti-inflammatory agent in dry eye disease [16]. Chlorosis-associated antigen (CsA), a protein derived from a fungus, has anti-inflammatory and immunopregnant properties by diminishing T-cell activation and reducing the synthesis of inflammatory cytokines. The molecule has a neutral charge and possesses hydrophobic properties, resulting in limited solubility in aqueous solutions. The method of action encompasses heightened tear production, reduced inflammation cytokine release and the safeguarding of conjunctival epithelial cells.

CsA is a calcineurin inhibitor that prevents T-cell infiltration, activation and subsequent production of inflammatory cytokines, hence modulating the immune response. The pharmacological substance exhibits the ability to infiltrate the cytoplasm of T-cells, facilitating subsequent interaction with cyclophilin. Hence, the formation of the CsA/cyclophilin complex hinders the dephosphorylation of the nuclear factor of activated T-cells (NFAT) and the transcription of cytokine genes (IL-2 and IL-4) mediated by calcineurin. Furthermore, CsA has been found to impede the activation of p38 and c-Jun N-terminal protein kinase. This leads to heightened synthesis of IL-2. The primary method by which CsA improves symptoms of dry eye illness is through its impact on T-cells. Furthermore, CsA has protective effects on conjunctival epithelial cells through apoptotic mechanisms, enhances the density of goblet cells on the conjunctival surface and promotes corneal integrity through immunomodulatory mechanisms. It inhibits the permeability of mitochondria and the opening of their pores, hence inhibiting the activation of the Fas ligand and caspase [17].

One of the pathogenetic causes of dryness in the eyes with dry keratoconjunctivitis is the hyperexcitability of cold thermoreceptors. All sensory neurons of the eye are classified into polymodal nociceptor neurons and selective mechanoreceptor neurons. Of all sensory neurons on the surface of the eye, cold receptors account for ~14%. At constant temperatures close to the surface temperature of the eye (34–35 °C), the vast majority of cold corneal thermoreceptor fibres constantly generate action potentials, the activity of which will increase with moderate cooling and decrease with heating. The onset of dry eye illness is attributed to the disruption of cold corneal thermoreception. In a study conducted by Gyenes *et al*. [6], the researchers examined the impact of CsA administration on the heightened activity of cold corneal nerve terminals in an animal model of dry eye accompanied by tear shortage. The study determined that CsA has an impact on the abnormal functioning of cold thermoreceptors, balancing their heightened activity. Additionally, it demonstrates immunomodulatory properties, hinders the growth of axons, decreases the expression of neurotrophin and stimulates the production of nerve growth factors by activating the p38 mitogen-activated protein kinase NFAT5 axis in the human epithelial cell line. The administration of cyclosporine systemically half an hour after damage was found to exhibit a neuroprotective effect. The observed enhancement in axon healing can be attributed to the suppression of white blood cell activity and the prevention of calcium-dependent cellular injury. Due to the subcutaneous administration of low doses of CsA, an increase in fibre density was observed. Neuroprotective effects on damaged peripheral nerves have also been described with topical application of CsA.

CsA 0.05% topical drops are approved by the United States Food and Drug Administration for use twice daily. Also, as a result of studies, it was noted that using CsA improved the Schirmer test, corneal fluorescein staining and increased goblet cell density [16]. The use of the drops resulted in beneficial outcomes such as enhanced tear production, decreased levels of inflammatory markers, heightened tear osmolarity, reduced epithelial cell death and the restoration of ocular conjunctival goblet cell abundance [17,19]. According to the findings of J. Kim *et al*. [20], the ocular penetration of CsA droplets is around 5%, mostly attributed to its limited solubility in water and its substantial molecular dimensions. Their study has described the effects of using new pH-sensitive contact lenses based on cellulose acetate phthalate filled with CsA. It is continuously released from the presented contact lenses at a temperature of 37 °C and a pH of 5.4 without an initial splash. A special feature of the presented lenses is the prevention of drug leakage during transportation and storage due to their response to the pH of the medium. Such lenses contain an optimal volume of water, provide the necessary amount of light and oxygen to the eye, are safe for cytotoxicity and do not irritate the eye surface. However, the disadvantage is the lack of sufficient evidence and the necessary human studies.

A large-scale study was conducted to compare various commercial forms of CsA. Restasis is a preservative-free anionic nanoemulsion; the solvent is castor oil, the emulsifier is polysorbate 80 and the stabilizer is carbomer. The advantage of this pharmaceutical preparation is the improvement of the general condition and the reduction of subjective symptoms (dryness and discomfort in the eyes). Zirun is a drug that uses new micelles as nanowires for drug delivery; its effect is five times higher than that of the conventional drug CsA. This drug is preferable for improving the performance of the Schirmer test (that is, it is a highly effective drug for restoring the normal functioning of the lacrimal system). All dosage forms of CsA cause certain adverse reactions expressed to varying degrees, but it was examined that the hydrophilic substance ethylene oxide used in Clacier creates a nanoemulsion with small particles of the same size, which leads to a reduction in eye irritation. TJ Cyporin was identified to be effective in improving tear film breakdown time [7].

Another registered form of CsA is Ikervis, a 0.1% sterile CsA emulsion without preservatives. These eye drops contain cetalkonium chloride, which increases the residence time of eye drops on the surface of the eye. Ikervis has an anti-inflammatory effect on the lacrimal glands, conjunctiva and eye surface. It was noted that this drug was better tolerated by older patients; they had substantially fewer side effects compared to younger patients [21,22]. Side effects when using regular 0.05% CsA eye drops are as follows: blurred vision, hyperaemia of the eye, itching of the eye and feeling of an alien body in the eye. Therefore, it may be inferred that CsA formulations exhibit efficacy and safety in the management of ocular dryness. Today, there are many different pharmacological forms of this drug, which differ in the concentration, composition of excipients and duration of stay on the eyes’ surface. Accordingly, the cost, availability, prevalence and risks of side effects differ. Therefore, it is necessary to individually select drugs for each patient, considering the severity of the disease, age and an appropriate assessment of possible risks of side effects.

The observed improvements in Schirmer test scores and tear film breakup time indicate a positive response to the treatment, suggesting that the use of CsA led to an increase in tear production and an enhancement in the stability of the tear film. These changes, while statistically significant, may also be clinically relevant as they reflect improvements in eye lubrication and protection, which are crucial factors in alleviating the symptoms of dry eye disease. However, to establish the clinical significance more robustly, it would be necessary to evaluate whether these improvements translate into meaningful symptom relief and better overall patient quality of life.

Comparison of the efficacy of CsA with other forms of this drug, such as Restasis and Zirun, showed significant benefits in improving the Schirmer test results and reducing subjective symptoms such as dryness and discomfort in the eyes. The sensitivity and specificity of the therapy were analysed using several clinical tools, including the Schirmer test, Oxford scale and visual analogue scale, all of which demonstrated significant improvements in patients treated with CsA. The results of these tests confirmed its effectiveness in increasing tear production, improving ocular surface health and reducing dryness symptoms. Additionally, comparisons with other products, such as Ikervis, which contains preservative-free CsA, showed similar anti-inflammatory effects, especially among older patients who tolerated the drug better and had fewer side effects. To further confirm the validity of the results, additional studies are needed to improve CsA delivery methods and reduce the likelihood of side effects, in particular by improving its bioavailability.

This study sought to make an impactful contribution to the understanding of the efficacy of CsA in the treatment of ocular surface diseases, particularly dry eye. The contribution was made by describing in detail the clinical outcomes and improving objective measures such as tear film breakup time, Schirmer test and Oxford scale scores, which demonstrated significant improvement in patients treated with this drug.

## Discussion

To provide scientifically based comparisons of the effectiveness of 0.05% CsA with other treatment approaches, a control group was introduced that received standard dry eye treatment, such as artificial tears or medications that reduce inflammation or moisturize the surface of the eye. This group allows us to evaluate not only the effectiveness of 0.05% CsA compared to other methods but also to track the potential benefits and limitations of each method for patients with different degrees of dry eye severity. The control group is important to increase the reliability of the results, as it allows us to assess whether the observed improvements are really due to the use of CsA and not to other factors, such as spontaneous symptom relief or the placebo effect. In addition, the inclusion of a control group allows us to determine differences in treatment efficacy between different therapeutic approaches, which may be important for developing optimal treatment plans for patients with dry eye. The use of a control group that received standard treatment allows for an objective comparison of the efficacy of 0.05% CsA with other methods, which increases the scientific credibility of the study and helps to determine the best approach to treating dry eye.

In the study conducted by D.C. Branisteanu *et al*. [23], the positive effect of using topical CsA therapy in pemphigoid scarring was described. A characteristic feature of ocular scarring pemphigoid is the development of bilateral chronic autoimmune recurrent conjunctivitis, which often leads to substantial visual impairment or blindness due to scarring of the conjunctiva. The incidence is one to six cases per ten thousand people, more often developing in the elderly. The primary manifestations of pemphigoid scarring and dry eye illness exhibit similarities, including sensations of burning, pruritus, a perception of an ocular foreign object and discomfort. Notably, when using correctly selected systemic therapy, unpleasant symptoms disappear in 90% of all cases. The drugs of choice with proven effectiveness are corticosteroids, tear substitutes and CsA drops.

Researchers L. Bartalena and M.L. Tanda [24] investigated the efficacy of cyclosporine in Graves’ orbitopathy. This pathology is an autoimmune disease of the orbit; it is an extrathyroid manifestation of Graves’ disease. Patients have increased levels of thyroid hormones, which cause inflammation in the tissues of the orbit. Also, due to a violation of the normal functioning of the eyelids and, accordingly, incomplete blinking, there is an uneven distribution of tears over the surface of the eye and its uneven evaporation. Common symptoms include a feeling of sand in the eyes, photophobia, eye pain, decreased colour perception, exophthalmos, diplopia and vision loss. Therewith, the average age of patients is ~50 years. The research included individuals with an average age of 51.6 years, representing the working-age population that was impacted. That is why the search for effective treatment is constantly underway. Currently, cyclosporine is recognized as one of the recommended drugs. It is an immunosuppressive agent that simultaneously affects humoral and cellular immunity. In a study involving 40 patients with moderate to severe Graves’ orbitopathy, it was determined that the use of combination therapy with cyclosporine and prednisone is more effective and helps to reduce relapses compared with prednisone monotherapy.

Researchers L. Qian and W. Wei [25] and C. Coles-Brennan *et al*. [26] looked into the endogenous factors that contribute to the development of dry eye disease. The aforementioned conditions encompass autoimmune and systemic illnesses, metabolic problems and genetic diseases. Sjögren’s syndrome is a persistent autoimmune condition characterized by an imbalance of lymphocytes and macrophages infiltrating the salivary and lacrimal glands. In individuals with this condition, there is an elevation in the levels of ILs (1, 6, 8) and TNF in the tear film. Multiple sclerosis is an autoimmune disorder characterized by the loss of myelin in the central nervous system [27]. As a result, sensory impulse conductivity decreases, leading to insufficient tear formation. Systemic disease xerophthalmia is caused by vitamin A deficiency. This vitamin is responsible for the differentiation and proliferation of conjunctiva and corneal epithelium. Hereditary familial dysautonomia (or Riley-Day syndrome) is an autosomal recessive disorder that disrupts the development of specific sensory and autonomic neurons during embryogenesis. Patients do not develop tears at the basal, reflex and emotional levels without proper treatment.

Changes in hormone concentrations affect the lacrimal and Meibomian glands. Testosterone is responsible for regulating lacrimal gland genes. Androgens regulate the secretion of fluid and lacrimal gland protein through steroid-specific receptors in acinar and ductal epithelial cells [28,29]. Low androgen levels are associated with lacrimal gland dysfunction and a lack of watery fluid. Reduced concentration of 5*a*-dihydrotestosterone leads to deterioration of the activity of the Meibomian glands; this contributes to the formation of an unstable film and an increase in the rate of tear evaporation. Notably, the participants of the presented study did not identify hereditary, autoimmune and systemic diseases that could cause dry eye disease in the selected group of patients (information about the health status of patients was obtained from the medical documentation provided by them and based on the collected anamnesis of life and illness). The exogenous cause of dry eye disease is digital eye strain [30]. This condition is characterized by visual impairment and a feeling of discomfort in the eyes; it is associated with using digital devices. The causes of digital eye strain are as follows: nearsightedness, farsightedness, astigmatism, accommodation abnormalities, convergence disorders, reduced blinking frequency, wearing contact lenses, poor indoor lighting, small screen size and fine print [31]. The main symptoms include frontal head pain, eye pain, diplopia, excessive eye strain, red eyes, blurred vision, lacrimation and back and neck pain.

Contact lens use is a significant contributing factor to the development of digital eye strain [32,33]. C. Coles-Brennan *et al*. [26] reported that among computer users, 83% of men and 87% of women who wear contact lenses had one or more symptoms of dry eyes. It is characteristic that the symptoms were more pronounced in those who used digital devices for more than 5 h a day. Notably, ~75% of the participants in the presented study noted that they used digital devices for ~4.5–5 h a day. Therewith, 52% of them wore contact lenses in everyday life. Given the large-scale prevalence of digital eye strain, researchers X. Ouyang *et al*. [34] and P.P. Choo *et al*. [35] proposed effective treatments and prevention for this condition. First, the ergonomic use of digital devices involves the implementation of many measures, such as using high-quality lighting and configuring visual settings, including resolution, text size, contrast and brightness. Second, when working with a computer/tablet, one should take breaks for 20 s every 20 min. It is also effective to use computer glasses that correct astigmatism, colour filters, eye drops and hints on screens to increase the frequency of blinks.

The treatment for dry eye condition in this trial was the use of 0.05% CsA eye drops. Therewith, a number of auxiliary non-pharmacological agents are presented in the articles of J. Sheppard *et al*. [36], Z.A.I.Y Hasan [37] and A. Song *et al*. [38]. The process of thermal pulsation of the Meibomian gland involves the application of heat and pulsing pressure to the region of both eyelids to dilute and eliminate the pre-existing contents. Indications for use are complicated forms of dry eye disease and chronic cystic diseases of the eyelids. During the use of intense pulsed light, photothermolysis occurs (a light wave of a certain length is used to destroy damaged blood vessels); it is indicated for telangiectasia, which can lead to the development of dry eye disease. Low-level light therapy is based on the effect of photobiomodulation (irradiation with non-ionizing light to stimulate the production of ATP by the Meibomian glands, which leads to endogenous healing) [39,40]. Microblepharoexfoliation is indicated in the treatment of tick infestations, *Demodex* and related blepharitis, which can lead to the development of dry eye disease. The essence of the technique is to clean the edge of the eyelids and eyelashes with a micro-sponge to remove the film, pathogens and toxins. The activation of the nasolacrimal reflex by hardware vibration stimulation of the external nasal nerve leads to an increase in tear production.

Compared to the aforementioned methods, the use of 0.05% CsA proved to be more effective in improving objective measures such as Schirmer’s test, tear film breakdown time and Oxford scale scores, confirming its direct effect on reducing inflammation and improving tear film stability. These results highlight the significant pharmacological benefits of CsA, as it acts by modulating the immune response and reducing the activation of inflammatory cytokines, which are key contributors to dry eye disease. This mechanism not only helps alleviate symptoms such as discomfort and dryness but also enhances the overall function of the ocular surface. In contrast, the additional non-pharmacologic treatments, while beneficial in improving dry eye symptoms, often serve as complementary therapies rather than standalone solutions. Methods such as thermal pulsation, intense pulsed light and low-level light therapy are effective in targeting specific aspects of the disease, such as improving meibomian gland function or addressing blood vessel damage, but they generally require a more comprehensive approach for optimal effectiveness. These techniques are primarily indicated for more complicated or chronic cases of dry eye disease, or they are often used in conjunction with pharmacological agents like CsA to provide more holistic care.

It is recommended to increase the humidity in the room, monitor daily hygiene of the eye and adjacent structures, not touch the eye mucosa with dirty hands and take frequent breaks when working with computers to improve the general condition of patients with dry eye disease. Sufficient quality sleep, proper nutrition, walking and sports are important. These recommendations are effective both for the treatment and prevention of diseases of the eye surface.

## Conclusions

As a result of the study, it was concluded that the use of 0.05% CsA eye drops for dry eye disease in the group under study led to a decrease in discomfort and pain and an improvement in objective indicators. According to the visual analogue scale, the following results were obtained: 6.8 points before the start of the study, 5.1 points on day 21 and 3.7 points on day 60. Objectively, there was an improvement in the time of destruction of the tear film (3.9 s – day 0, 4.6 s – day 21 and 6.8 s – day 60, respectively); improvement in the Schirmer test (4 mm – day 0, 4.9 mm – day 21 and 6.3 mm – day 60); indicators on the Oxford scale (day 0 – 77% – 1 point and 23% – 2 points; day 21 – 78% – 1 point and 22% – 2 points; day 60 – 80% – 1 point and 20% – 2 points). During the study, 20% of patients complained of moderate side effects from using drops (pain during instillation of the eye and redness of the eye surface).

Diseases of the surface structures of the eye are common and have a diverse aetiology (exposure to exogenous toxins, violation of personal hygiene, excessive use of digital devices and the presence of autoimmune, systemic, hereditary and hormonal disorders). The following is recommended to increase the effectiveness of treatment for eye diseases: daily walks in the fresh air, healthy sleep and food, work in high-quality lighting, setting image parameters on digital devices and using hints on computer screens to increase the frequency of blinking.

CsA is a peptide that is obtained from fungus and has anti-inflammatory and immunomodulatory properties. It effectively diminishes the activation of T-lymphocytes and the synthesis of inflammatory cytokines. The method of action encompasses heightened tear production, reduced inflammation cytokine release and the safeguarding of conjunctival epithelial cells. When applied topically, no severe side effects were detected (the possible individual intolerance and the development of anaphylactic shock are notable). However, a substantial disadvantage of eye drops is their low bioavailability. Therefore, further randomized clinical trials aimed at improving the delivery of CsA to eye structures are recommended.

## Abbreviations

CsA: cyclosporine A
NFAT: nuclear factor of activated T-cells
